# Multidimensional Scaling Applied to Histogram-Based DNA Analysis

**DOI:** 10.1155/2012/289694

**Published:** 2012-07-24

**Authors:** António C. Costa, J. A. Tenreiro Machado, Maria Dulce Quelhas

**Affiliations:** ^1^Department of Informatics Engineering, Institute of Engineering, Polytechnic of Porto, Rua Dr. António Bernardino de Almeida 431, 4200-072 Porto, Portugal; ^2^Department of Electrical Engineering, Institute of Engineering, Polytechnic of Porto, Rua Dr. António Bernardino de Almeida 431, 4200-072 Porto, Portugal; ^3^National Health Institute and Biochemical Genetics Unit, Institute of Medical Genetics Center Jacinto de Magalhães, Praça Pedro Nunes 88, 4099-028 Porto, Portugal

## Abstract

This paper aims to study the relationships between chromosomal DNA sequences of twenty species. We propose a methodology combining DNA-based word frequency histograms, correlation methods, and an MDS technique to visualize structural information underlying chromosomes (CRs) and species. Four statistical measures are tested (Minkowski, Cosine, Pearson product-moment, and Kendall *τ* rank correlations) to analyze the information content of 421 nuclear CRs from twenty species. The proposed methodology is built on mathematical tools and allows the analysis and visualization of very large amounts of stream data, like DNA sequences, with almost no assumptions other than the predefined DNA “word length.” This methodology is able to produce comprehensible three-dimensional visualizations of CR clustering and related spatial and structural patterns. The results of the four test correlation scenarios show that the high-level information clusterings produced by the MDS tool are qualitatively similar, with small variations due to each correlation method characteristics, and that the clusterings are a consequence of the input data and not method's artifacts.

## 1. Introduction

DNA related information can be analyzed in many different ways, including by methods based on “word frequency” histograms derived from DNA sequences [1]. Histograms are a condensed representation of the original information and allow further processing methods, like correlation, which are not viable in the original data. The correlation between histograms can be computed, producing a correlation matrix that can serve as input to other methods for high-level information extraction and tabular/graphical analysis like the multidimensional scaling (MDS) technique, which is able to create low-dimensional representations of complex data while preserving similarities between data points. In [2], the authors describe how the Kendall *τ* rank correlation method [3] is used to generate the correlation matrix and how a Multidimensional Scaling (MDS) tool [4] is able to generate three-dimensional representations of spatial and structural relationships of the chromosomes and species. In that paper, only one correlation method is applied to the generation of correlation matrices, but many other correlation methods exist and can be used for studying chromosomal/species relationships. As such, we compare and evaluate a set of correlation methods in order to determine if those relationships show up in all methods and are similar. Our main goals are to find out if, for each of several correlation methods and word lengths used in the processing of DNA sequences,

- the MDS tool generates three-dimensional representations featuring spatial and structural patterns;
- its spatial and structural patterns denote significant differences for distinct correlation methods;
- the results from MDS tool are qualitatively similar, independently of the correlation method used.

It should be noted that important contributions in this topic [1, 5] were proposed using alignment-free sequence comparison methods, but the proposed method is based on different concepts.

Bearing these ideas in mind, this paper is organized as follows.  Section 2 presents the biological concepts and mathematical tools, formulating its application in the context of DNA sequence decoding.  Section 3 analyzes the correlation between CRs and several species, by investigating data representation using MDS applied to twenty species and their CRs. Finally, Section 4 outlines the main conclusions and open issues.

## 2. Mathematical Tools and DNA Decoding

The chromosomal DNA code of the twenty species was downloaded from the DNA sequence database of the University of California Santa Cruz Genome Bioinformatics site [6]. In each CR, repeated strings of more than 12 symbols were previously masked and replaced by “N” symbols, in order to ignore the nongenomic and nonregulatory sequence data. In consequence, we are handling an alphabet composed of symbols, namely, {T, C, A, G, N}. In terms of DNA data, an option was made for a set of twenty species, aiming to explore the dynamic analysis by changing the sequence length *n* in the range 1 ≤ *n* ≤ 8. The twenty species include eleven mammals {Hu, Ch, Or, Rm, Po, Eq, Ox, Dg, Rn, Mm, Op}, two birds, Chicken and Zebra finch {Ga, Tg}, two fishes, Zebrafish and Tetraodon {Zf, Tn}, two insects, Gambiae mosquito and Honeybee {Ag, Am}, two nematodes, *Caenorhabditis elegans* and *Caenorhabditis briggsae *{Ce, Cb}, and one fungus, Yeast {Sc}, with a grand total of *p* = 421 CRs. The characteristics of chosen species and its DNA are presented in Table 1.

The DNA implements an alphabet composed by the symbols {T, C, A, G}. Any simple translation to a numerical counterpart may impose bias and destroy intrinsic information. Consequently, it was decided to directly process the non-numerical code. Due to the immense volume of information, a histogram-based measure was adopted. Nevertheless, in general, histograms do not capture dynamics. In order to overcome this limitation, a flexible pattern detection algorithm based on counting the sequence of symbols was considered [1]. By “flexible” we mean that the algorithm can count sequences of length *n* items, each one composed by one of the four base symbols.

With the exception of Yeast (Sc), the available CR data includes a fifth symbol (“N”), corresponding to masked DNA symbols not belonging to the genome, which typically appear in large contiguous sequences. For example, in the Human Y CR file there are 59373566 base pairs, of which 33710000 are “N” (56.78%) arranged in 17 sequences, the largest one with 30000000 symbols. Another example is the Chicken Ga25 CR, with 2051775 base pairs, of which 663879 are “N” (32.67%) arranged in 274 sequences, the largest one with 500000 symbols. HoY and Ga25 are just two examples of CRs with a percentage of “N” symbols greater than 10%, but most of the CRs have smaller percentages.

We decided not to use “N” in sequences as a fifth symbol or not to replace it by any of the symbols {T, C, G, orA}, because that would introduce an unknown bias in the sequence processing. We then considered the following two approaches:

- remove all “N” symbols in a preprocessing step or,
- process sequences but ignoring any sequence with an “N”.

Although (a) and (b) may seem different, we concluded that differences were minimal and that (a) could be advantageously used without compromising the quality of results and conclusions.

Using as examples {Hu, Ck, Tn, Ag} nuclear CRs, and a sequence length of *n* = 8 in Table 2, the rightmost column synthesizes the differences for the (a) and (b) approaches. For Ga25 the Pearson correlation coefficient *r* between (a) and (b) sequences with length *n* = 8 yields *r* > 0.9999717, while for HoY the corresponding coefficient *r* is >0.9999999. We conclude that both approaches are statistically equivalent for the envisaged DNA decoding. Therefore, we opted to discard the “N” symbol before histogram construction.

We have different statistics when considering the length ranging from *n* = 1, representing merely a static counting of *m* = 4^1^ states, up to *n* = 8, representing a system with *m* = 4^8^ (65536) states. During the bin counting two possible approaches may be considered, namely, windows without any overlapping and windows with a partial overlapping of the *n* base sequence. Therefore, two extreme opposite cases were tested, namely, successive counting windows with zero and with *n* − 1 adjacent bases in the DNA. In the first case, for a DNA strand of length *L* and sequences of length *n*, results a total of approximately *L*/*n* counting windows, while for the second it yields *L* − *n* + 1 counting windows. Previous tests revealed that both schemes lead to similar qualitative results, with some slight differences in the smaller CRs [2]. In order to get a more robust counting, we adopted the one-base sliding window (i.e., overlapping of *n* − 1 consecutive bases).

Having generated the histograms, the second step in the analysis consists in evaluating their similarities by means of suitable correlation indices. In this study, we evaluate four correlation methods [3, 7, 8], namely, the Minkowski *r*_*ij*_^*M*^, Cosine *r*_*ij*_^*C*^, Pearson product-moment *r*_*ij*_^*P*^, and Kendall *τ* rank *r*_*ij*_^*K*^ as given by

(1) $$\begin{array}{c} r_{ij}^{M}={\left[\sum_{r=1}^{m}{\left|f_{i}\left(r\right)-f_{j}\left(r\right)\right|}^{\alpha }\right]}^{1/\alpha },\;\alpha >0, \end{array}$$

(2) $$\begin{array}{c} r_{ij}^{C}=\frac{\sum_{r=1}^{m}f_{i}\left(r\right)f_{j}\left(r\right)}{\sqrt{\sum_{r=1}^{m}{\left[f_{i}\left(r\right)\right]}^{2}\sum_{r=1}^{m}{\left[f_{j}\left(r\right)\right]}^{2}}}, \end{array}$$

(3) $$\begin{array}{c} r_{ij}^{P}=\frac{m\sum_{r=1}^{m}f_{i}\left(r\right)f_{j}\left(r\right)-\sum_{r=1}^{m}f_{i}\left(r\right)\sum_{r=1}^{m}f_{j}\left(r\right)}{\sqrt{m\sum_{r=1}^{m}{\left[f_{i}\left(r\right)\right]}^{2}-{\left[\sum_{r=1}^{m}f_{i}\left(r\right)\right]}^{2}}\sqrt{m\sum_{r=1}^{m}{\left[f_{j}\left(r\right)\right]}^{2}-{\left[\sum_{r=1}^{m}f_{j}\left(r\right)\right]}^{2}}}, \end{array}$$

(4) $$\begin{array}{c} r_{ij}^{K}=\frac{\left(\text{number of concordant pairs}\right)-\left(\text{number of disconcordant pairs}\right)}{\left(1/2\right)m\left(m-1\right)}, \end{array}$$

where *f*_*i*_(*r*) and *f*_*j*_(*r*) represent the relative frequencies of histograms *i* and *j* for bin *r* and *m* denotes the total number of bins. If (*f*_*i*_, *f*_*j*_) represents a set of joint observations from two variables, any pair of observations are said to be concordant (discordant) if the ranks for both elements agree (disagree), while for identical rank the pair is neither concordant nor discordant.

For the purpose of visualizing the correlation results, the multidimensional scaling (MDS) technique is adopted [9–11]. The MDS is a mathematical tool that represents, in a low-dimensional map, a set of data points whose similarities (or, alternatively, distances) are defined in a higher dimensional space by means of a symmetric matrix **S** = [*s*_*ij*_]. In case of similarities (or, alternatively, distances) and classical MDS, the main diagonal is composed of ones (or, alternatively, zeros), while the rest of the matrix elements must obey the restriction 0 ≤ *s*_*kl*_ ≤ 1 (*s*_*kl*_ ≥ 0), *k*, *l* = 1,…, *p*, where *p* is the total number of cases under comparison [12]. It should be noted that MDS works with relative measurements. Therefore, MDS maps are not sensitive to translations or rotations. The axes have only the meaning and units (if any) of the measuring index and packages usually apply a heuristic procedure for centering the chart. In practical terms, this means that MDS maps are analyzed on the basis of proximity of (or, alternatively, distance between) points and comparison of the resulting “cloud” of points. Usually, in order to improve the graphical representation, 2-D and 3-D MDS plots are used and its consistency verified by means of Shepard and/or stress charts [13].

## 3. Analysis of DNA Sequence Histograms

In this section, we start by analyzing a limited part of the global information by means of direct methods. We verify some limitations due to the huge volume of data. This fact motivates the adoption of a more efficient visualization method, namely, the MDS, that is applied to the complete set of data.

### 3.1. Analysis of Six Species Using a Diagram Visualization Method

In this subsection, we compare six mammals, namely, Human, Common Chimpanzee, Orangutan, Rhesus monkey, Pig, and Opossum, denoted by {Ho, Ch, Or, Rm, Pi, andOp}. In this preliminary analysis, it is adopted that *n* = 8 in the histogram construction and the correlation expression (2), leading to a 6 × 6 matrix **S** with considerable information. Considering a threshold value of 99.5% for selecting the “most similar CRs” (i.e., smaller values are ignored) we get the groups presented in Figure 10. We observe that some CRs with distinct numbering are very similar as, for example, Rm16 is clearly correlated with Hu17, Ch17, and Or17, while others are very different from the rest, such as, for example, HuY, ChY, Rm19, Pi12, and OpX. In terms of species we conclude that:

- Hu has twenty CRs correlated in the first place with Ch and two with Or,
- Ch has eighteen CRs correlated in the first place with Hu and six with Or,
- Or has twenty one CRs correlated in the first place with Ch and three with Hu,
- Rm has one CR correlated in the first place with Hu and zero with Ch and Or,
- Pi and Op have zero CRs correlated with the rest of the species.

Therefore, we conclude that Ch is the species closest to the Hu, Rm is far from the trio {Hu, Ch, Or}, and {Pi, Op} have no proximity with the rest.

**Figure 1:**
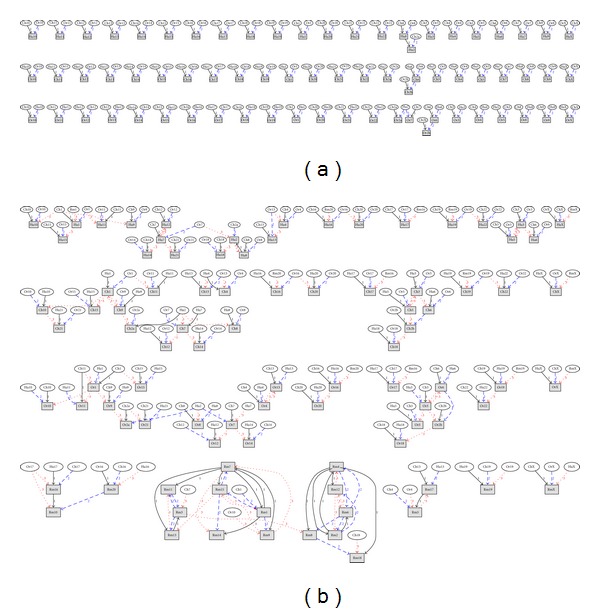
The *r* most correlated CRs for the group {Hu, Ch, Or} (a) *r* = 3, (b) *r* = 4. Legend: grey rectangle: objective CR (arrows point towards him), ellipse: CR that correlates with another edge *r* : *r*th most correlated CR.

This information can be depicted graphically. Figure 1 shows visualization graphs generated by Graphviz [14], an open-source software for representing structural information as diagrams of abstract graphs and networks. The *r* = {3, 4} most correlated CRs for the group {Hu, Ch, Or} reveals clearly, for example, triplets of CRs 19, 20, and 22, groups of CRs 13 and 4, groups of CRs 16, 17, and Rm20.

**Figure 2:**
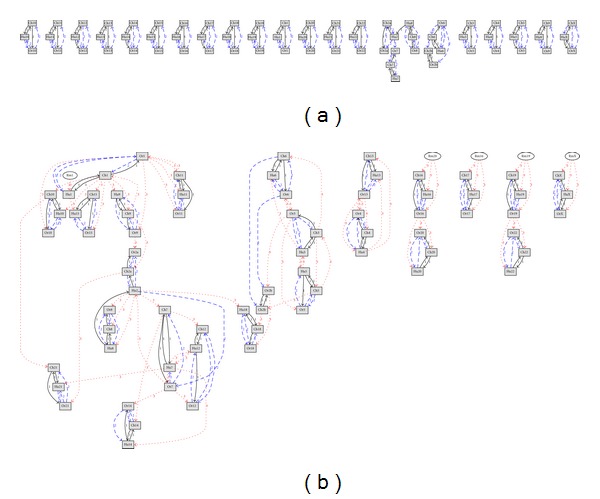
The *r* most correlated CRs for the group {Hu, Ch, Or} (a) *r* = 2, (b) *r* = 3. Legend: Grey rectangle: objective CR (arrows point towards him), ellipse: CR that correlates with another edge *r* : *r*th most correlated CR.

For the trio {Hu, Ch, andOr}, Figure 2 shows the chart for the cases of *r* = 2 and *r* = 3.

These tests reveal that even for a limited set of data directed graph methods lead to complicated representations.

### 3.2. Analysis of Twenty Species Using the MDS Visualization Method

**Figure 3:**
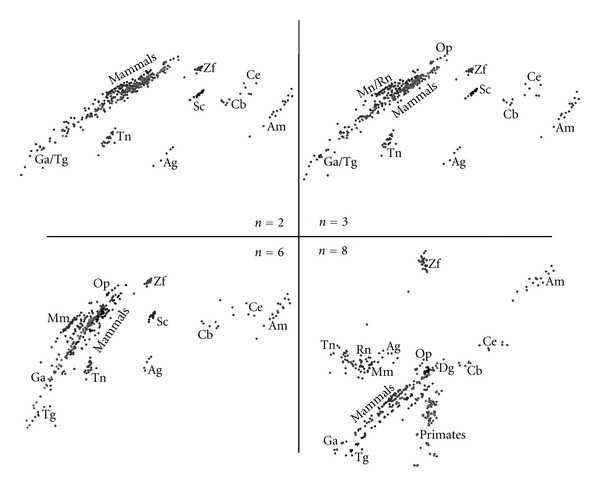
The 2-dimensional MDS plots, for DNA sequence lengths *n* = {2, 3, 6, 8} and the Minkowski correlation *r*_*ij*_^*M*^ with *α* = 2.

**Figure 4:**
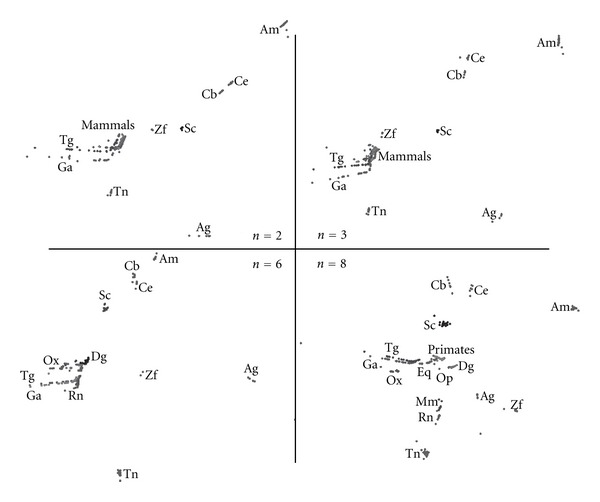
The 2-dimensional MDS plots, for DNA sequence lengths *n* = {2, 3, 6, 8} and the Cosine correlation *r*_*ij*_^*C*^.

**Figure 5:**
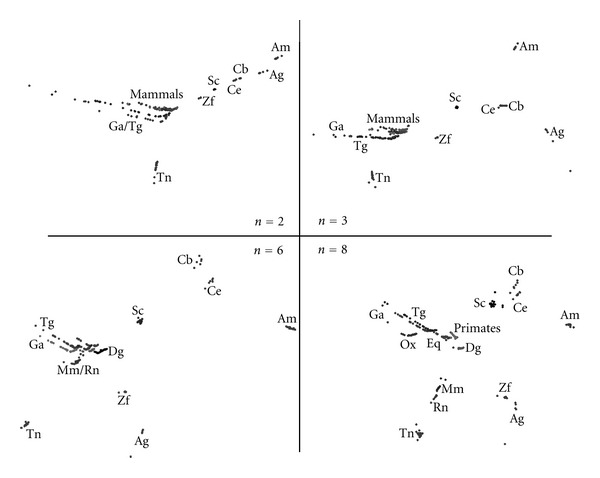
The 2-dimensional MDS plots, for DNA sequence lengths *n* = {2, 3, 6, 8} and the Pearson product-moment correlation *r*_*ij*_^*P*^.

**Figure 6:**
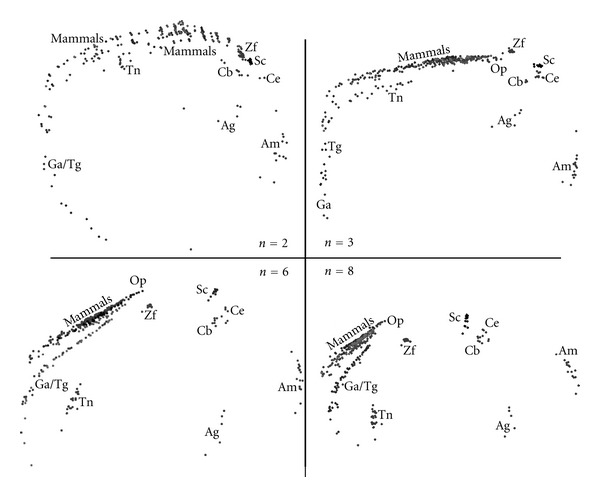
The 2-dimensional MDS plots, for DNA sequence lengths *n* = {2, 3, 6, 8} and the Kendall *τ* rank correlation *r*_*ij*_^*K*^.

In this subsection, we compare the complete set of species using the MDS method. Therefore, after computing all the chromosomal histograms of the twenty species for 1 ≤ *n* ≤ 8, the GGobi package [4] is used for generating the MDS plots corresponding to the correlation methods described in (1)–(4). In Figures 3 to 6, we depict MDS plots, using a classical metric dissimilarity analysis, for each correlation method when *n* = {2, 3, 6, 8}. The choice for the aforementioned values of n was motivated by the following considerations:

- *n* = 2; it is the smallest value of *n* for reasonably discriminating DNA-based frequency histograms;
- *n* = 3; the protein coding machinery in CRs uses triplets (3) of bases to specify amino acids [15];
- *n* = 6; a larger value of *n* that is also multiple of 3 and potentially sensitive to the protein coding mechanisms;
- *n* = 8; a larger value of *n* that is not multiple of 3 and is computationally tractable.

The MDS maps for the remaining values of *n* are not depicted due to space limitation. Values of *n* > 8 were not considered because they impose an increasingly greater computational burden: the number of frequency bins in a histogram is *m* = 4^*n*^, each correlation depends on *m* ^2^ operations and a complete correlation study requires approximately *p* ^2^/2 correlations.

Figure 3 presents MDS plots for the Minkowski correlation *r*_*ij*_^*M*^ revealing the emergence of chromosomal patterns for all values of *n*. We note that the MDS plots vary progressively and smoothly from *n* = 2 up to *n* = 8. When *n* = 8, we observe that mammals' CRs are more spatially separated and that the primates' CRs “diverge” from the rest of the mammals. The Minkowski correlation depends on the value of the parameter *α* = 0. For *α* = 1 and *α* = 2, it yields the commonly known Manhattan (or City) and Euclidean distances, while for the limiting case *α* → *∞* we obtain the Chebyshev distance. After testing the MDS plots for several values of *α*, *α* = 2 was adopted as representative of this method.

Figure 4 presents MDS plots for the cosine correlation *r*_*ij*_^*C*^ demonstrating clear chromosomal patterns. Again we conclude that the MDS plots evolve from *n* = 2 up to *n* = 8. Moreover, mammals' CRs become more separated as *n* reaches larger values such as *n* = 6 and *n* = 8. It is clearly noticeable that the MDS plots in Figures 3 and 4 are geometrically very distinct but depict chromosomal patterns and structures that lead to conclusions of the same type. This visual effect is common in MDS maps, namely, with the conclusions being drawn in relative terms rather than in an absolute perspective, with the patterns and not the absolute coordinates of points being important.

Figure 5 presents MDS plots for the Pearson product-moment correlation *r*_*ij*_^*P*^. Again, chromosomal patterns are clearly observable for all values of *n* and the smooth evolution from *n* = 2 up to *n* = 8. We note that the Pearson correlation method is based on the product of moments, which means that it is invariant to separate changes in location and scale of the two histogram sequences. The MDS plots of Figure 5 also depict chromosomal patterns and structures, but geometrically distinct from the MDS plots represented in previous figures.

Finally, Figure 6 presents MDS plots for the Kendall *τ* rank correlation *r*_*ij*_^*K*^ leading to similar conclusions. 

Comparing the four indices {*r*_*ij*_^*M*^, *r*_*ij*_^*C*^, *r*_*ij*_^*P*^, *r*_*ij*_^*K*^} that feed the MDS plots, we conclude that the Kendal *τ* correlation *r*_*ij*_^*K*^ reveals more distinct transitions between MDS plots and, consequently, the chart for *r*_*ij*_^*K*^ and *n* = 8 seems to be the one that depicts more noticeable chromosomal patterns and geometrical structures.

A standard assessment tool in MDS analysis is the Shepard plot, which provides a qualitative measure of the goodness of fit. Considering *i* and *j* the row and column indexes of matrix **S**, the Shepard plot represents the dissimilarities **D**_*ij*_ against the fitted distances **b**_*ij*_ = 〈**x**_*i*_i, **x**_*j*_〉 (where 〈·, ·〉 represents the inner product for classical scaling), or the residuals *Re*s_*ij*_ = *f*(**D**_*ij*_) − **b**_*ij*_ (where*f*(**D**_ij_) is the transformation of dissimilarities and is a power for metric scaling). In terms of MDS qualitative analysis in this paper, the goodness of fit is very high for all values of *n* and all types of correlation methods. Being the MDS quantitative assessment described by the stress value, Figure 5 depicts the stress plots for the Kendall *τ* correlation method and the limit cases of *n* = 2 and *n* = 8, showing the usual monotone decreasing shape. For other correlation methods, the stress plots show a similar behavior.

**Figure 7:**
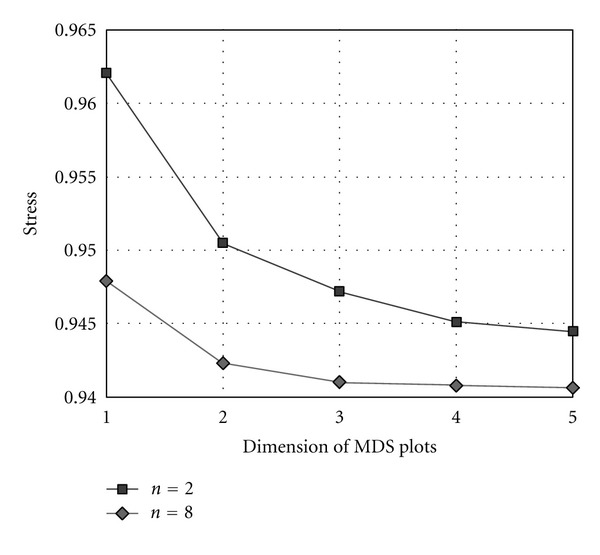
Stress plots of the MDS results generated by the Kendall *τ* rank correlation method for DNA sequence lengths of *n* = 2 and *n* = 8.

**Figure 8:**
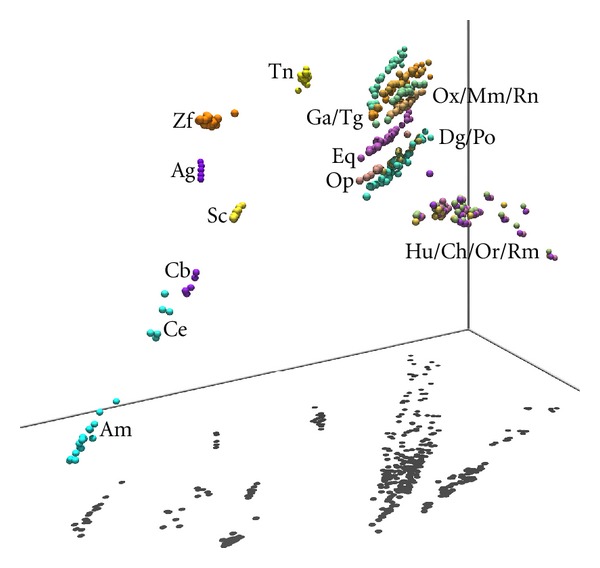
The 3-dimensional rendering of the MDS plot for *n* = 8 and the Minkowski correlation *r*_*ij*_^*M*^ with *α* = 2.

**Figure 9:**
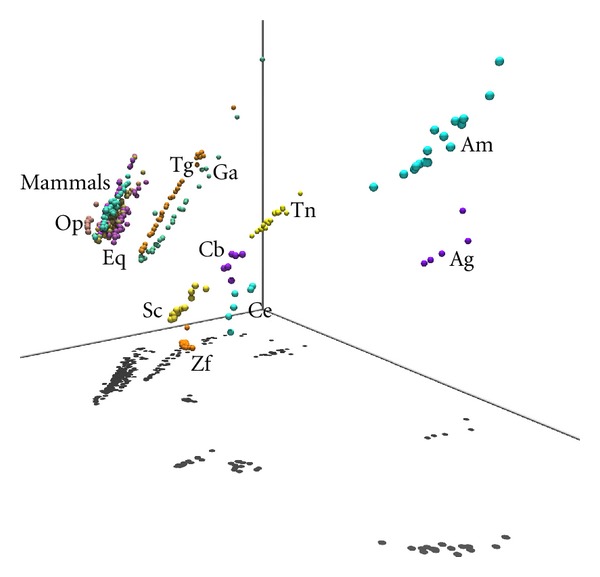
The 3-dimensional rendering of the MDS plot for *n* = 8 and the Kendall *τ* rank correlation *r*_*ij*_^*K*^.

**Figure 10:**
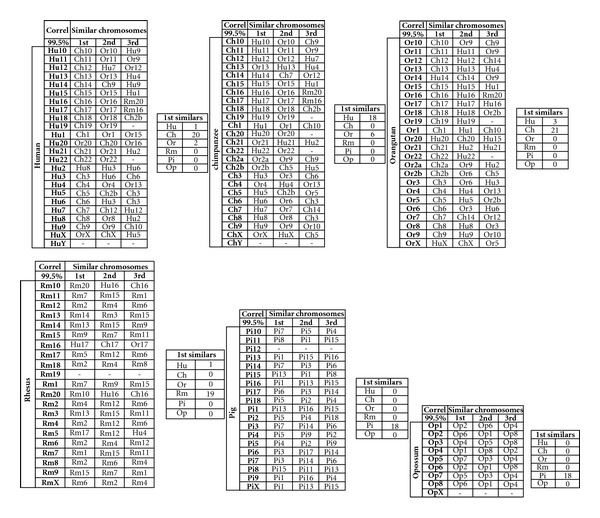
Chromosome similarities for the groups {Hu, Ch, Or, Rm, Pi, Op} using *n* = 8 and a threshold value of 99.5%.

Although the chart of Figure 7 supports the adequacy of adopting a two-dimensional representation for the MDS output, it also shows that a three-dimensional map can lead to a slightly improved rendering of MDS plots. In this line of thought, Figures 8 and 9 show two “visually enhanced” three-dimensional MDS maps for *n* = 8, corresponding to the Minkowski index *r*_*ij*_^*M*^ with *α* = 2 and the Kendall *τ* correlation *r*_*ij*_^*K*^. Both figures include visual cues (like perspective effects, shadows on objects/on the floor, and three coordinate axis) to help in the spatial and structural understanding of chromosomal relationships.

In Figure 8, it is clearly noticeable that a primate species' cluster near to a mammals' cluster is having next to it the aves' cluster. The mammals and aves' cluster depict a “linear” disposition of CRs, which is confirmed by the corresponding shadows on the floor. A “linear” chromosomal disposition is also observed in species like {Ag, Am, Sc}, but not in species like fishes {Zf, Tn} and nematodes {Cb, Ce}. It is also noteworthy to mention the “parallelism” between the linear dispositions of the mammal species (excluding primates) and the aves {Ga, Tg}.

In Figure 9, we can observe that mammal species are organized in a cluster, all of them depicting a “linear” spatial disposition. The aves {Ga, Tg} also cluster together, near the mammals, each one with a clear “linear” disposition. The shadows over the floor (a visual cue) help understanding these spatial and structural relationships. For the remaining species, the fishes {Zf, Tn}are spatially far apart, only Tn depicting a “linear” spatial disposition. This same disposition somewhat exists in the {Am, Ag, Sc}, but not in the nematodes {Cb, Ce}.

As mentioned in Section 2, MDS works with data that characterized the relative distance between the objects. Therefore, in MDS maps, rotation and translation have no special meaning and user can adopt the one that is more useful to visualize the clusters. Identically, MDS charts with different number of points or with distinct measuring indices cannot be compared, neither with each other, nor in the perspective of the coordinates of the points. Therefore, a “good” MDS representation is simply the one that adopts a measuring technique that for the phenomenon under study and for the number of objects leads to a map where user can visualize easily clusters that make sense for that particular application. In this line of thought in this paper, the association of several correlation measures for the 421 CRs proved to lead to a comprehensive pattern easily visualized and assertively interpretable under the light of present-day knowledge.

In this study, the nuclear genomic information used is still incomplete, as explained in [6]. For many of the species referred in Table 1, there is a considerable amount of nuclear DNA sequence data not yet attached to CRs or with an unknown placement. This undesirable uncertainty may contribute to misleading results, not caused by the mathematical and computational tools adopted. While the focus of this paper was mainly an interspecies comparison, the same methodology can be used for revealing intraspecies chromosomal patterns. We also foresee the application of the described methodology to the study of mitochondrial DNA sequences. These issues will be the subject of further research.

## 4. Conclusions

Chromosomes have a code based on a four-symbol alphabet and the information can be analyzed with mathematical tools usually adopted in the analysis of complex systems [16]. In this paper, it was applied a histogram-based conversion scheme for establishing a numerical signal and the resulting information was studied by means of four distinct correlation measures. The application to the CRs of twenty species, with a grand total of 421 CRs, revealed that the combination of sequence lengths of eight symbols, the Kendall *τ* rank correlation, and the MDS visualization is the most promising one, leading to the emergence of patterns that can be easily and assertively interpreted and compared.

The three-dimensional patterns of CRs depicted in Figures 6 and 7 “point” to a high level of genomic structuring in each species (“linear” and “spherical” arrangements) and between species (“parallelism” between mammals and aves). Although we do not have an immediate explanation for this noticeable multidimensional structuring, it may be related to higher levels of information structure underlying CRs.

## Figures and Tables

**Table 1:** Main characteristics of the twenty species and their chromosomes.

Species	Tag	Group	Chromosomes
Human	Hu	mammal	1 2 3 4 5 6 7 8 9 10 11 12 13 14 15 16 17 18 19 20 21 22 X Y
Chimpanzee	Ch	mammal	1 2a 2b 3 4 5 6 7 8 9 10 11 12 1 14 15 16 17 18 19 20 21 22 X Y
Orangutan	Or	mammal	1 2a 2b 3 4 5 6 7 8 9 10 11 12 13 14 15 16 17 18 19 20 21 22 X
Rhesus	Rm	mammal	1 2 3 4 5 6 7 8 9 10 11 12 13 14 15 16 17 18 19 20 X
Pig	Po	mammal	1 2 3 4 5 6 7 8 9 10 11 12 13 14 15 16 17 18 X
Opossum	Op	mammal	1 2 3 4 5 6 7 8 X
Horse	Eq	mammal	1 2 3 4 5 6 7 8 9 10 11 12 13 14 15 16 17 18 19 20 21 22 23 24 25 26 27 28 29 30 31 X
Dog	Dg	mammal	1 2 3 4 5 6 7 8 9 10 11 12 13 14 15 16 17 18 19 20 21 22 23 24 25 26 27 28 29 30 31 32 33 34 35 36 37 38 X
Ox	Ox	mammal	1 2 3 4 5 6 7 8 9 10 11 12 13 14 15 16 17 18 19 20 21 22 23 24 25 26 27 28 29 X
Mouse	Mm	mammal	1 2 3 4 5 6 7 8 9 10 11 12 13 14 15 16 17 18 19 X Y
Rat	Rn	mammal	1 2 3 4 5 6 7 8 9 10 11 12 13 14 15 16 17 18 19 20 X
Chicken	Ga	ave	1 2 3 4 5 6 7 8 9 10 11 12 13 14 15 16 17 18 19 20 21 22 23 24 25 26 27 28 W Z
Zebra finch	Tg	ave	1a 1b 1 2 3 4 4a 5 6 7 8 9 10 11 12 13 14 15 17 18 19 20 21 22 23 24 25 26 27 28 Z
Zebra fish	Zf	fish	1 2 3 4 5 6 7 8 9 10 11 12 13 14 15 16 17 18 19 20 21 22 23 24 25
Tetraodon	Tn	fish	1 2 3 4 5 6 7 8 9 10 11 12 13 14 15 16 17 18 19 20 21
Mosquito	Ag	insect	2l 2r 3l 3r X
Honey bee	Am	insect	1 2 3 4 5 6 7 8 9 10 11 12 13 14 15 16
*C. elegans*	Ce	worm	1 2 3 4 5 X
*C. briggsae*	Cb	worm	1 2 3 4 5 X
Yeast	Sc	fungus	1 2 3 4 5 6 7 8 9 10 11 12 13 14 15 16

(Note: CRs, Ga32, and Tg16 were ignored due to their very small base pair count).

**Table 2:** Influence of symbol “N” upon the statistics.

Chromosome	Sequences with “N” removed (*α*)	Sequences with “N” filtered (*β*)	((*α* − *β*/*β*) in %
Ga25	1367889	1366030	0.136088%
Ga3	110204947	110177075	0.025297%
Tn1	20304845	20315377	0.051869%
Tn15	6235253	6236842	0.025484%
AgX	21470369	21477782	0.034527%
Ag2l	48065434	48071405	0.012423%
HoY	25653559	25653447	0.000437%
Ho5	177695253	177695218	0.000020%
